# Gene polymorphisms in RANKL/RANK/OPG pathway are associated with ages at menarche and natural menopause in Chinese women

**DOI:** 10.1186/s12905-015-0192-3

**Published:** 2015-04-13

**Authors:** Peng Duan, Zhi-Ming Wang, Jiang Liu, Li-Na Wang, Zhi Yang, Ping Tu

**Affiliations:** Department of Endocrinology and Metabolism, Nanchang Key Laboratory of Diabetes, The Third Hospital of Nanchang/The Affiliated Nanchang Hospital of Southern Medical University, NO.2, South Xiangshan road, Nanchang city, Xihu District, Jiangxi province 330009 People’s Republic of China

**Keywords:** RANKL, RANK, OPG, *TNFSF11*, *TNFRSF11A*, *TNFRSF11B*, Polymorphism, Age at menarche, Age at natural menopause

## Abstract

**Background:**

Age at menarche (AAM) and age at natural menopause (AANM) have been shown intimately associated with woman’s health later in life. Previous studies have indicated that AAM and AANM are highly heritable. RANKL/RANK/OPG signaling pathway is essential for mammary gland development, which is also found associated with post-menopausal and hormone-related diseases. The aim of this study was to evaluate associations between the polymorphisms in the *TNFSF11, TNFRSF11A* and *TNFRSF11B* genes in the RANKL/RANK/OPG pathway with AAM and AANM in Chinese women.

**Methods:**

Post-menopausal Chinese women (n = 845) aged from 42 to 89 years were recruited in the study. Information about AAM and AANM were obtained through questionnaires and the genomic DNA was isolated from peripheral blood from the participants. Total 21 tagging single nucleotide polymorphisms (SNPs) of *TNFSF11*, *TNFRSF11A* and *TNFRSF11B* were genotyped.

**Results:**

Three SNPs of *TNFRSF11A* (*rs4500848*, *rs6567270* and *rs1805034*) showed significant association with AAM (*P* < 0.01, *P* = 0.02 and *P* = 0.01, respectively), and one SNP (*rs9962159*) was significantly associated with AANM (*P* = 0.03). Haplotypes TC and AT (*rs6567270-rs1805034*) of *TNFRSF11A* were found to be significantly associated with AAM (*P* = 0.01 and *P* = 0.02, respectively), and haplotypes GC and AC *(rs9962159-rs4603673*) of *TNFRSF11A* showed significant association with AANM (*P* = 0.03 and *P* < 0.01, respectively). No significant association between *TNFSF11* or *TNFRSF11B* gene with AAM or AANM was found.

**Conclusions:**

The present study suggests that *TNFRSF11A* but not *TNFSF11* and *TNFRSF11B* genetic polymorphisms are associated with AAM and AANM in Chinese women. The findings provide evidence that genetic variations in RANKL/RANK/OPG pathway may be associated with the onset and cessation of the menstruation cycle.

## Background

Age at menarche (AAM) and age at natural menopause (AANM) have been shown intimately associated with woman’s health later in life. Women with early menarche have high risks of breast cancer [1], ovarian cancer [2], type 2 diabetes [3] or metabolic syndrome [4], whereas late menarche can increase the risk of osteoporosis [5]. On the other hand, early AANM is associated with increased risk of cardiovascular diseases [6] and osteoporosis [7]. Recent data have shown that AAM and AANM were associated with all-cause mortality [8]. However, the factors that affect AAM and AANM are not entirely clear.

AAM and AANM are complex traits which are influenced by both genetic and environmental factors and their interactions [9]. Twin and familial studies have indicated that AAM and AANM are highly heritable, ranging from 45% to 74% for AAM [10,11] and from 49% to 87% for AANM [12,13]. Genes involved in hormone biosynthesis and metabolic pathways were found to be associated with AAM and AANM [14,15], however, no specific genes have been identified yet.

The receptor activator of nuclear factor-kappa B ligand (RANKL), its receptor RANK and the decoy receptor osteoprotegerin (OPG) belong to the tumor necrosis factor superfamily and they are encoded by genes *TNFSF11*, *TNFRSF11A* and *TNFRSF11B*, respectively. RANKL/RANK/OPG signaling pathway plays important roles in bone modeling and remodeling [16], cell death and proliferation, inflammation, and immunity [17,18]. RANKL/RANK/OPG pathway is also found associated with post-menopausal and hormone-related diseases, such as osteoporosis [19] and reproductive cancer [20]. Furthermore, RANKL is found to be essential for mammary gland development in mice by promoting proliferation and maintaining survival of mammary epithelial cells [21]. Mammary gland changes are one of the hallmarks during menarche and menopause [22,23]. Therefore, RANKL/RANK/OPG pathway may involve in modulating the onset and cessation of the menstrual cycle. The present study investigated the associations of single nucleotide polymorphisms and haplotypes in *TNFSF11*, *TNFRSF11A* and *TNFRSF11B* genes in RANKL/RANK/OPG pathway with AAM and AANM in Chinese females.

## Methods

### Participants

A total 1026 post-menopausal women from ten community centers in Nanchang from December 2011 to December 2012 were enrolled in the study. All the participants were from Han Chinese ethnic group. Age at interview, AAM, AANM, detailed medical history, birth history (number of live delivery), and abortion information (number of abortions) were obtained through a self-designed questionnaire, all the information collected in the study was self-reported. AAM was defined as the age at the first menstrual period. AANM was defined as one year without menstruation after the age at the last menstrual period. For each participant, height (cm) and weight (kg) were measured. The body mass index (BMI) was calculated as weight/height^2^.

All of the participants were subjected to blood counts, liver and kidney function tests, fasting plasma glucose tests. Subjects included in the study had normal blood counts, normal liver and kidney functions and blood glucose levels. Subjects were excluded from the study if they suffered from diseases and surgeries that could affect menstruation, such as severe chronic diseases, rheumatic diseases (e.g. systemic lupus erythematosus, rheumatoid arthritis), severe endocrine and metabolic diseases (e.g. diabetes, hyperparathyroidism, pituitary or adrenal diseases), malabsorption diseases (e.g. chronic diarrhea, anorexia nervosa), cancer, and uterine or ovarian resection. Participants who had taken glucocorticosteroid or sex hormone within the past 3 months were also excluded. Finally, 845 subjects were included in the study. The study was approved by the Ethics Committee of The Third Hospital of Nanchang. Written informed consent was obtained from every participant.

### TagSNP selection

Tagging SNPs of the three genes were selected from the software program Haploview version 4.2 [24] (http://www.broad.mit.edu/mpg/haploview/) with minor allele frequencies (MAF) > 10% in the Chinese Han population in HapMap (http://www.hapmap.org/), and the pairwise linkage disequilibrium (LD) was greater than a threshold of r^2^ (r^2^ = 0.8). In addition to, SNPs reported in previous studies or potentially functional SNPs in three candidate genes were forced into the SNP selection process. Finally, a total of 21 SNPs were selected in three genes (9 in *TNFRSF11A* gene, 6 in *TNFSF11* gene, and 6 in *TNFRSF11B* gene). Of these, 18 SNPs are located in the introns of the three genes, one in 5′-UTR, two in the exonic region. All of these SNPs were authenticated using the NCBI (http://www.ncbi.nlm.nih.gov/SNP/) and HapMap databases.

### Genotyping

Approximately 5 mL of venous blood was collected from all of the participants after a minimum of 10 h fasting and stored in tubes containing 100 μL of 10% ethylene diaminetetraacetic acid (EDTA). Genomic DNA was extracted from whole blood samples using the QIAamp DNA Mini Kit (Qiagen Inc., Hilden, Germany). DNA samples concentration and quality were detected spectrophotometrically at 260/280 nm and stored at −80°C until analysed. Genotyping was performed using the high-throughput Sequenom genotyping platform (MassARRAY MALDI-TOF MS system, Sequenom Inc., San Diego, CA). For quality control, 5% of the samples were repeatedly genotyped, and the results were found to be 100% concordant.

### Statistical analyses

Genotype frequencies and concordance of the SNPs were analyzed for the Hardy-Weinberg equilibrium (HWE) using the *χ*^2^ test. Data were expressed as mean ± standard deviation. The stepwise multiple regression analysis was used to analyze the relationships between the SNPs and AAM and AANM, subsequently, each SNP with different genotype was analyzed independently using one-way univariate analysis of variance (ANOVA), BMI, age at interview, number of deliveries and abortions were considered as covariates and were adjusted during analysis. Bonferroni correction was used to adjust the *P* values for multiple comparisons. The statistical analyses were performed using SPSS version 13.0 for Windows (SPSS Inc., Chicago, IL, USA). The linkage disequilibrium structure and allele frequencies were examined using Haploview 4.2 software [24]. The significance of each haplotype within the defined blocks was analyzed by PLINK software [25] (http://pngu.mgh.harvard.edu/~purcell/plink/). All analyses were two-tailed, and *P* -value < 0.05 was considered statistically significant.

## Results

### Characteristics of the study participants

The basic characteristics of the 845 participants aged from 42 to 89 years were shown in Table 1. The mean age at interview was 60.88 ± 8.72 years, the mean AAM was 14.97 ± 2.00 years and AANM was 48.77 ± 4.16 years. No statistically significant association was observed between AAM and AANM (*P* = 0.15).

**Table 1:** **Characteristics of the 845 participants**

**Characteristics**	**Average**	**95% CI**
*Age (years)*	60.88 ± 8.72	60.30-61.47
*Age at menarche (years)*	14.97 ± 2.00	14.83-15.10
*Age at menopause (years)*	48.77 ± 4.16	48.49-49.05
*Height (cm)*	154.18 ± 5.97	153.78-154.58
*Weight (kg)*	58.21 ± 8.73	57.62-58.80
*BMI (kg/m* ^*2*^ *)*	24.46 ± 3.20	24.25-24.68
*Number of deliveries*	2.14 ± 1.32	2.05-2.23
*Number of spontaneous abortions*	0.13 ± 0.44	0.10-0.16
*Number of induced abortions*	1.21 ± 1.27	1.13-1.30

The data are presented as the means ± standard deviation. BMI, body mass index. CI, confidence interval.

### SNP genotyping and linkage disequilibrium

**Figure 1:**
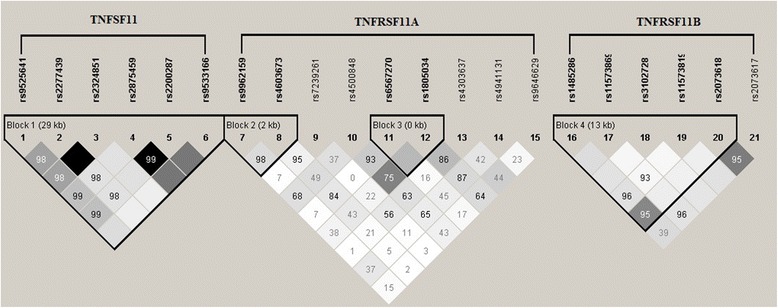
Linkage disequilibrium (LD) patterns for *TNFSF11, TNFRSF11A* and *TNFRSF11B* genes. LD plots with *r* ^2^ values were generated by Haploview software. R^2^ values are indicated by the degree of darkness, increasing from white to black. The *D’* values multiplied by 100 are shown as numbers in the diamonds. There are one in *TNFSF11* (block 1), two blocks in *TNFRSF11A* (block 2 and block 3), and one in *TNFRSF11B* (block 4), respectively.

The basic characteristics of the SNPs are listed in Table 2. All study SNPs had a minor allele frequency of at least 0.1 and were in agreement with Hardy-Weinberg equilibrium (*P* > 0.05). Linkage disequilibrium between alleles at polymorphic loci was shown in Figure 1. Four haplotype blocks and seventeen of the most common haplotypes (frequency > 5%) were further analyzed for the association of haplotype with AAM and AANM.

**Table 2:** **Associations for the SNPs of** ***TNFSF11, TNFRSF11A*** **and** ***TNFRSF11B*** **genes with AAM and AANM**

**Gene**	**SNP**	**Allele**	**Function**	**HWE**	**MAF**	**AAM**			**AANM**		
						**Beta**	***P***	***P*** **-Bonf**	**Beta**	***P***	***P*** **-Bonf**
*TNFRSF11B*	*rs1485286*	*C/T*	*Intron*	0.4237	T = 0.406	0.0697	0.49	1.00	0.2428	0.25	1.00
*TNFRSF11B*	*rs11573869*	*A/G*	*Intron*	0.8783	G = 0.165	−0.0529	0.69	1.00	0.1109	0.68	1.00
*TNFRSF11B*	*rs3102728*	*T/C*	*Intron*	0.2913	C = 0.138	0.0726	0.61	1.00	−0.2352	0.43	1.00
*TNFRSF11B*	*rs11573819*	*G/A*	*Intron*	0.9402	A = 0.157	0.0501	0.71	1.00	−0.1709	0.54	1.00
*TNFRSF11B*	*rs2073618*	*C/G*	*Asn by Lys*	0.5148	G = 0.258	−0.0065	0.95	1.00	0.2687	0.25	1.00
*TNFRSF11B*	*rs2073617*	*A/G*	*UTR-5*	0.7887	G = 0.382	−0.1137	0.26	1.00	0.1741	0.41	1.00
*TNFSF11*	*rs9525641*	*T/C*	*Intron*	0.1546	C = 0.472	0.0810	0.42	1.00	−0.0656	0.75	1.00
*TNFSF11*	*rs2277439*	*A/G*	*Intron*	0.5905	G = 0.295	−0.0611	0.56	1.00	0.2430	0.27	1.00
*TNFSF11*	*rs2324851*	*G/A*	*Intron*	0.6728	A = 0.294	−0.0699	0.51	1.00	0.2351	0.29	1.00
*TNFSF11*	*rs2875459*	*C/T*	*Intron*	0.8542	T = 0.220	−0.1036	0.38	1.00	−0.0189	0.94	1.00
*TNFSF11*	*rs2200287*	*G/A*	*Intron*	0.8891	A = 0.220	−0.0898	0.44	1.00	−0.0215	0.94	1.00
*TNFSF11*	*rs9533166*	*T/C*	*Intron*	0.5125	C = 0.131	−0.1694	0.23	1.00	−0.2154	0.47	1.00
*TNFRSF11A*	*rs9962159*	*A/G*	*Intron*	0.4172	G = 0.435	0.1338	0.17	1.00	−0.4434	0.03	0.57
*TNFRSF11A*	*rs4603673*	*C/G*	*Intron*	0.7190	G = 0.162	−0.1363	0.31	1.00	−0.2551	0.36	1.00
*TNFRSF11A*	*rs7239261*	*C/A*	*Intron*	0.2342	A = 0.239	−0.1656	0.14	1.00	0.3123	0.18	1.00
*TNFRSF11A*	*rs4500848*	*C/T*	*Intron*	0.7267	T = 0.262	−0.3395	<0.01	0.04	−0.0378	0.87	1.00
*TNFRSF11A*	*rs6567270*	*T/A*	*Intron*	0.1116	A = 0.408	0.2265	0.02	0.39	0.1197	0.55	1.00
*TNFRSF11A*	*rs1805034*	*T/C*	*Ala by Val*	0.3758	C = 0.288	−0.2790	0.01	0.22	0.0069	0.98	1.00
*TNFRSF11A*	*rs4303637*	*C/T*	*Intron*	0.9818	C = 0.471	0.1618	0.10	1.00	0.0701	0.73	1.00
*TNFRSF11A*	*rs4941131*	*T/C*	*Intron*	0.8166	C = 0.330	0.0437	0.67	1.00	−0.1892	0.38	1.00
*TNFRSF11A*	*rs9646629*	*G/C*	*Intron*	0.2416	C = 0.460	0.1461	0.14	1.00	−0.2256	0.28	1.00

HWE, *P* values for Hardy-Weinberg equilibrium. MAF, minor allele frequency. Beta, the regression coefficient. *P*-Bonf, *P*-value by Bonferroni correction. AAM, age at menarche. AANM, age at natural menopause.

### Association analyses of the SNP and haplotypes with AAM and AANM

Three SNPs in *TNFRSF11A*, i.e. *rs4500848*, *rs6567270* and *rs1805034*, showed significant association with AAM (*P* **<** 0.01, *P* = 0.02 and *P* = 0.01, respectively), whereas only *rs9962159* in *TNFRSF11A* was significantly associated with AANM (*P* = 0.03) (Table 2). After correction of age at interview, BMI, number of deliveries and abortions, the associations between those SNPs and AAM or AANM were found significant. After the Bonferroni correction, the *rs4500848* was still significantly associated with AAM (*P* = 0.04), however, the associations between the others SNPs with AAM or AANM were no longer statistically significant (Table 2). Individuals with the T/T genotype of SNP *rs4500848* had an earlier onset of menarche by 0.59 years than did those with the C/C genotype. Likewise, women with the G/G genotype of SNP *rs9962159* had an earlier menopause by 0.79 years than those with the A/A genotype (Table 3).

**Table 3:** **Significant associations for the single SNPs of** ***TNFSF11, TNFRSF11A*** **and** ***TNFRSF11B*** **genes with AAM and AANM**

**Genotype**	**n**	**AAM**	**Genotype**	**n**	**AAM**
*rs4500848*			*rs1805034*		
*C/C*	471	15.16 ± 1.95	*C/C*	64	14.53 ± 2.17
*C/T*	313	14.76 ± 2.07	*C/T*	358	14.86 ± 2.04
*T/T*	61	14.57 ± 1.85	*T/T*	423	15.12 ± 1.93
*P-value*		<0.01	*P-value*		0.04
**Genotype**	**n**	**AAM**	**Genotype**	**n**	**AANM**
*rs6567270*			*rs9962159*		
*A/A*	152	15.35 ± 2.07	*G/G*	166	48.45 ± 4.18
*A/T*	385	14.92 ± 2.02	*G/A*	403	48.56 ± 4.23
*T/T*	308	14.84 ± 1.91	*A/A*	276	49.26 ± 4.00
*P-value*		0.03	*P-value*		0.05

Two haplotypes (TC and AT) of block *rs6567270-rs1805034* of *TNFRSF11A* were found significantly associated with AAM (*P* = 0.01 and *P* = 0.02, respectively) (Table 4). Haplotypes GC and AC of block *rs9962159-rs4603673* of *TNFRSF11A* were significantly associated with AANM (*P* = 0.03 and *P* **<** 0.01, respectively). Haplotype TAGCGT of block *rs9525641-rs2277439-rs2324851-rs2875459-rs2200287-rs9533166* of *TNFSF11* showed marginally significant association with AAM (*P* = 0.06). Notably, all the significantly associated SNPs and haplotypes were observed in *TNFRSF11A*. SNPs and haplotypes in *TNFSF11* and *TNFRSF11B* genes did not show significant association with either AAM or AANM.

**Table 4:** **The associations of haplotypes of** ***TNFSF11, TNFRSF11A*** **and** ***TNFRSF11B*** **genes with AAM and AANM**

**Gene**	**Haplotype**	**Frequency**	**AAM**		**AANM**	
			**Beta**	***P***	**Beta**	***P***
*TNFSF11*: *rs9525641-rs2277439-rs2324851-rs2875459-rs2200287-rs9533166*						
	*TAGTAC*	0.131	−0.1686	0.24	−0.2171	0.46
	*TAGTAT*	0.088	0.0148	0.93	0.2392	0.50
	*TGACGT*	0.292	−0.0737	0.49	0.1958	0.38
	*CAGCGT*	0.469	0.0773	0.44	−0.1007	0.63
	*TAGCGT*	0.017	0.7854	0.06	−1.3300	0.09
*TNFRSF11A*: *rs9962159-rs4603673*						
	*AG*	0.161	−0.1455	0.28	−0.2432	0.38
	*GC*	0.434	0.1294	0.18	−0.4383	0.03
	*AC*	0.405	−0.0578	0.55	0.5747	<0.01
*TNFRSF11A* : *rs6567270-rs1805034*						
	*TC*	0.288	−0.2787	0.01	0.0069	0.98
	*AT*	0.408	0.2263	0.02	0.1197	0.55
	*TT*	0.305	−0.0113	0.91	−0.1408	0.51
*TNFRSF11B* : *rs1485286-rs11573869-rs3102728-rs11573819-rs2073618*						
	*TATGG*	0.250	0.0311	0.78	0.2791	0.24
	*CATAC*	0.155	0.0770	0.57	−0.1808	0.52
	*CACGC*	0.139	0.0726	0.61	−0.2352	0.43
	*CGTGC*	0.164	−0.0407	0.76	0.1067	0.70
	*TATGC*	0.154	0.0955	0.48	0.0410	0.88
	*CATGC*	0.130	−0.2041	0.17	−0.1828	0.56

The analyses were performed under an additive model adjusted for age at interview and BMI. Beta, regression coefficient.

## Discussion

According to the previous studies, there was a direct relationship between AAM and AANM, women with earlier menarche had earlier menopause in Poland [26]. However, other studies had reported no association between AAM and AANM [27]. In this tudy, no statistically significant association was observed between AAM and AANM. The present study revealed that three SNPs (*rs4500848*, *rs6567270* and *rs1805034*) and two haplotypes of *TNFRSF11A* showed significant association with AAM in Chinese women. These findings are in line to a previous report by Pan et al. [28], the authors found five SNPs (*rs7239261*, *rs8094884*, *rs3826620*, *rs8089829*, and *rs9956850*) and seven haplotypes of *TNFRSF11A* significantly associated with AAM in Chinese women. Thus, polymorphisms in *TNFRSF11A* are highly associated with AAM in Chinese women. In contrast to *TNFRSF11A*, SNPs of *TNFSF11* did not show association with AAM in our study. Noticeably, two SNPs (*rs9525641* and *rs2200287*) of *TNFSF11* displayed a strong association with AAM in white women [29], but no significant association was observed between the two SNPs and AAM in our study. The inconsistency between the results of the present study and the other [29] may due to the different ethnic populations used, different sample sizes and statistical approaches. The inconsistent results were also observed in the association of *TNFSF11* gene polymorphisms and AANM. In the present study, no significant association between polymorphisms of *TNFSF11* and AANM was found in Chinese women, however, such relationship was reported in white women, two SNPs (*rs346578* and *rs9525641*) of *TNFSF11* showed association with AANM [29]. Regardless of the discrepancy in *TNFSF11*, we and the others [29] both found a strong association between polymorphisms of *TNFRSF11A* and AANM.

The associations between polymorphisms of *TNFRSF11A* with AAM and AANM found in the present study can be explained by its possible roles in mammary gland development and menstruation. First, *TNFRSF11A* belongs to RANKL/RANK/OPG signaling pathway. RANKL plays an important role in mammary gland development, indicating its potential role in regulating or responding to sex hormone fluctuation and subsequently influencing menstrual cycles. Studies have shown that gonadotropin-releasing hormone (GnRH) can modulate RANKL expression in breast cancer cells [30], and expressions of RANK and RANKL in different cell lines are controlled by estrogen [31], follicle-stimulating hormone [32], and dehydroepiandrosterone [33]. Estrogen is also found to regulate gene expression and ratio of the *RANKL/OPG* [34]. Second, RANKL signaling pathway can stimulate ductal side-branching and alveologenesis in the mammary gland in mouse [35]. RANKL can be induced in mammary epithelium and can regulate the proliferation of cells [36]. Therefore, RANK signaling pathway may influence onset of puberty and menstrual cycle by regulating mammary gland development. Third, genome-wide association (GWA) studies have identified some novel genetic loci associated with AAM and AANM [37,38]. Gene set enrichment pathway analyses using the GWA dataset found that nuclear factor-kappa B (NF-κB) signaling pathway may be associated with timing of menopause [39]. Recent studies have revealed that NF-κB pathway plays an important role in mammary ductal morphogenesis [40], and ovarian cell function in animals [41]. It was well established that the RANKL/RANK/OPG pathway can activate NF-κB and its downstream players [42]. Thus, genes (e.g. *TNFRSF11A)* in the RANKL/RANK/OPG pathway may have role in the onset and cessation of the menstruation cycle.

The present study has some limitations. First, beside genetic other factors can influence timing of menarche and menopause, e.g. environment and socioeconomic status. We studied only the relationship between genetic variations and AAM and AANM. Furthermore, gene-environment interactions may also play a role in causing variation in the AAM and AANM. Second, The data of AAM and AANM were collected through retrospective self-report, which may cause recall bias. The participants in the study were aged from 42 to 89 years, with long interval periods, which might potentially incur recall error. It is reported that the accuracy of long-term recall of AAM and AANM varied from 70% to 84% [43,44]. In fact, it was found that some participants could not remember the exact age at the first menstrual period, and those subjects were excluded from the study. Large-scale studies are needed to confirm current findings, and the precise mechanisms underlying the observed associations in our study remain to be determined.

## Conclusions

The present study, for the first time, demonstrated that *TNFRSF11A* but not *TNFSF11* and *TNFRSF11B* genetic polymorphisms are associated with AAM and AANM in Chinese women. The findings provide evidence that genetic variations in RANKL/RANK/OPG pathway may be associated with the onset and cessation of the menstruation cycle.

## Abbreviations

AAM: Age at menarche
AANM: Age at natural menopause
OPG: Osteoprotegerin
RANK: Receptor activator of nuclear factor-kappa B
RANKL: Receptor activator of nuclear factor-kappa B ligand
SNP: Single nucleotide polymorphisms
BMI: Body mass index
HWE: Hardy-Weinberg equilibrium
GnRH: Gonadotropin-releasing hormone
GWA: Genome-wide association
NF-κB: Nuclear factor-kappa B
